# Toxicity Studies of Ethyl Maltol and Iron Complexes in Mice

**DOI:** 10.1155/2017/2640619

**Published:** 2017-01-19

**Authors:** Zhen Li, Jieli Lu, Chonghui Wu, Quanhai Pang, Zhiwei Zhu, Ruipeng Nan, Ruochen Du, Jia Chen

**Affiliations:** ^1^College of Animal Science and Veterinary Medicine, Shanxi Agricultural University, Taigu, Shanxi 030801, China; ^2^College of Life Science, Shanxi Agricultural University, Taigu, Shanxi 030801, China; ^3^Institute of Farm Product Storage and Fresh-Keeping, Shanxi Academy of Agriculture Science, Taiyuan 030031, China

## Abstract

Ethyl maltol and iron complexes are products of ethyl maltol and the iron found in the cooking pots used to prepare the Chinese dish, hot-pot. Because their safety is undocumented, the toxicity study of ethyl maltol and iron complexes was conducted in male and female Kunming (KM) mice. The animal study was designed based on the preliminary study conducted to determine the median lethal dose (LD_50_). The doses used in the study were 0, 1/81, 1/27, 1/9, and 1/3 of the LD_50_ (mg kg body weight (BW)^−1^ day^−1^) dissolved in the water. The oral LD_50_ of the ethyl maltol and iron complexes was determined to be 743.88 mg kg BW^−1^ in mice. The ethyl maltol and iron complexes targeted the endocrine organs including the liver and kidneys following the 90 D oral exposure. Based on the haematological data, the lowest-observed-adverse-effect level (LOAEL) of the ethyl maltol and iron complexes was determined to be 1/81 LD_50_ (9.18 mg kg BW^−1^ day^−1^) in both male and female mice. Therefore, we suggest that alternative strategies for preparing the hot-pot, including the use of non-Fe-based cookware, need to be developed and encouraged to avoid the formation of the potentially toxic complexes.

## 1. Introduction

**Figure 1:**
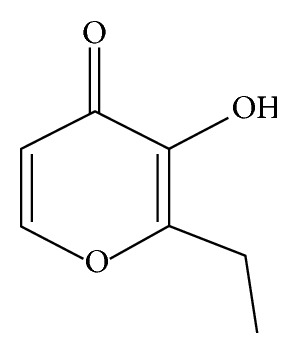
Ethyl maltol (C_7_H_8_O_3_, EMA).

Ethyl maltol (C_7_H_8_O_3_, Figure 1) is an important food additive and the main component of a type of incense added to food. It is widely used to enhance the flavour of commercial food products such as candy, cookies, chocolate, and beverages [1–3] and extend the product shelf-life, inhibit the growth of melanoma, and enhance film uniformity in beverages, food, cigarettes, cosmetics, pharmaceuticals, and photosensitive materials [4]. In previous studies, ethyl maltol was found to be approximately six times more potent than its homologue maltol, in improving the aroma and flavour of food [3].

Hot-pot is an original traditional Chinese dish, and its most important characteristic is its fragrance. Ethyl maltol is the key food additive and flavour enhancer that imparts the characteristic hot-pot fragrance. In the process of preparing hot-pot, the businesses involved often randomly add a drop of ethyl maltol-containing incense to the bottom of the cooking pots, which when fabricated with Fe can react with the steam produced during the boiling process to produce iron oxide (Fe_3_O_4_). In previous studies, ethyl maltol was confirmed to combine with Fe^3+^ in different ratios, namely, 1 : 1, 1 : 2, and 1 : 3 [5]. In the process of making the well-loved Chinese hot-pot dish more flavourful, the ethyl maltol inevitably comes in contact with the Fe to form iron complexes that are kinetically labile. Although the ethyl maltol enhances the scent of foods, a high dietary intake may lead to headaches, nausea, and vomiting and could affect the liver and kidney functions [2, 3, 6].

Gralla et al. [7] evaluated the biological safety of ethyl maltol following acute, subacute, and chronic administration, and their results suggested that ethyl maltol is safe, but their information on the toxicity of ethyl maltol and iron complexes was limited. Therefore, this present study was designed to evaluate the acute and subchronic toxicity of ethyl maltol and iron complexes, to provide information on their safety for addressing the clinical implications of their consumption.

## 2. Materials and Methods

### 2.1. Test Article

The ethyl maltol was purchased from Beijing Newt Biotechnology Development Co., Ltd, with a purity of 99.5% that was detected using the ultraviolet (UV)-2501 UV-visible (VIS) spectrophotometer. The deionized water used in the tests was prepared using the HB-0.25-200T equipment (China) while the distilled water was sterilized at 0.15 kPa at 121°C for 15 min.

To simulate hot-pot production, the ethyl maltol was completely dissolved in hot distilled water and boiled in an iron pan for 1 h to ensure the formation of the red-purple complexes, and then the mixture was brought to 25°C. The stability of the ethyl maltol and iron complexes was maintained for up to 1 day at room temperature, and they were prepared daily at 7 a.m.

### 2.2. Animals

The 70 and 100 Kunming (KM) mice of both sexes (6- and 4-week-old, resp.) used in this study were obtained from Shanxi Medical University (China). Following their arrival, the health condition of all the animals was examined to confirm their suitability for use in the study and they were allowed to acclimate to the laboratory environment for 5 days. The animals were housed by sex in groups of five per cage in an environment-controlled animal room and were provided with standard commercial diet and drinking water ad libitum. Except for minor variations, all the animal rooms were monitored and maintained under a 12 h light-dark cycle with temperature ranging from 20 to 25°C and relative humidity of 40%–60%. All the animal procedures performed in this study were reviewed and approved by the Animal Experimental Welfare and Ethical Inspection Committee of the Chinese Center for Disease Control and Prevention (Approval number IACUC#15-026).

### 2.3. Experimental Design

In a preliminary dose-finding study, 70 mice were stratified by weight and assigned randomly to seven groups of five males and five females each. The mice in the control and groups 1, 2, 3, 4, 5, and 6 were given the ethyl maltol and iron complexes at doses of 0, 425.98, 532.48, 665.6, 832, 1040, and 1300 mg kg BW^−1^, respectively, by gavage for 14 days at 9 a.m. daily. The mice were monitored to observe their general behaviour and signs of toxicity continuously for 3 h after the gavage. The mice were further observed once a day for up to 14 days for behavioural changes and signs of toxicity and death. The surviving mice were euthanized while those that died from the poisoning were necropsied. We calculated the median lethal dose (LD_50_) of the ethyl maltol and iron complexes selected from the study doses based on the value obtained.

One hundred mice of both sexes were stratified by weight and randomly divided into five groups of 10 males and 10 females each, which were given a mixture of water and the ethyl maltol and iron complexes, at single doses of 1/81, 1/27, 1/9, and 1/3 LD_50 _ mg kg BW^−1^ by gavage for 90 days. In addition, the control group was also given the same amount of distilled water (without ethyl maltol) boiled in the iron pan. The general cage-side observations for clinical signs were performed twice during the acclimation period, once daily after administration of the test article, and the detailed clinical observations were performed once daily. The animals were observed closely for any behavioural changes daily and were examined individually outside their home cage environments ensuring they were undisturbed. The body weights of the animals and food consumption were monitored for 10 days during the study period.

### 2.4. Measurement of Biochemical and Haematological Parameters

On day 45 (D45), the animals were fasted for 4 h, and blood samples were collected from their tail tips for haematological and biochemical analysis while 24 h urine samples were also collected. On D90, the animals were fasted for 4 h, and then their body weights were measured, and whole blood samples were collected while the 24 h urine samples were also collected. Then, the mice were euthanized and subsequently necropsied.

#### 2.4.1. Haematological Parameters

The blood samples were analysed using an automatic blood analyser (MEK-6318K, Japan) for the red blood cell (RBC) and white blood cell (WBC) counts, haemoglobin (Hb), and percentage of leukocytes including lymphocytes (L), neutrophil (N), monocyte count (M), eosinophil (E), and basophil (B) counts.

#### 2.4.2. Serum Biochemistry

The whole blood biochemistry of the mice including alanine amino transferase (ALT), aspartate amino transferase (AST), alkaline phosphatase (ALP), total bilirubin (TBIL), lactate dehydrogenase (LDH), total protein (TP), albumin (ALB), globulin (GLO), albumin/globulin ratio (A/G), blood urea nitrogen (BUN), and creatinine (Cr) was analysed. The blood samples were collected from the orbital artery of mice anesthetized with pentobarbital sodium by intraperitoneal injection, centrifuged at 3000 rpm for 10 min at 4°C, and subsequently analysed using an automatic chemical analyser (HITACHI7180, China).

### 2.5. Organ Weight and Histopathology

After the blood collection, the animals were euthanized, and the organs including the hearts, livers, spleens, kidneys, and lungs were harvested for histological examination. We also determined the absolute and relative organ weights (based on terminal body weights) of the heart, liver, spleen, kidneys, and lungs, which were calculated as follows:

(1) Relative organ weight %=Absolute organ weight gBody weight g×100.

The histopathological examinations were conducted on all the organs and the liver and kidneys tissues of all animals that showed obvious gross lesions were fixed in 10% buffered formalin for 24 h and then they were routinely processed for paraffin embedding [8].

### 2.6. Statistical Analysis

All the data are expressed as the means ± standard deviation (SD) and comparisons among the different groups were performed using an analysis of variance (ANOVA) and the IBM statistical package for the social sciences (SPSS) statistics 22 software was used for all the analysis. The significance level was set at 5 and 1% (*P* < 0.05 and *P* < 0.01, resp.). The LD_50_ value was determined according to the Bliss method [9].

## 3. Results

### 3.1. Preliminary LD_50_ Determination of Ethyl Maltol and Iron Complexes

Compared with the control group mice, the main behavioural signs of toxicity observed in the complex-treated mice were righting reflex disappearance, asthenia, and locomotor activity reduction (Table 1). The histological investigation showed different degrees of degeneration in the liver and kidneys cells, as well as oedema and haemorrhage in the liver and kidneys. No mortality was observed in 24 h, after which the mice in group 6 showed the first mortality followed by the other groups. After 1 week, no additional deaths were observed. LD_50_ of the ethyl maltol and iron complexes, which was evaluated using the Bliss method, was 743.88 mg/kg. According to the standard acute toxicity classification, the ethyl maltol and iron complexes belong to level 3 category, suggesting they possessed low toxicity.

### 3.2. Effect of Ethyl Maltol and Iron Complexes on Feed Intake and Growth of Mice

**Figure 2:**
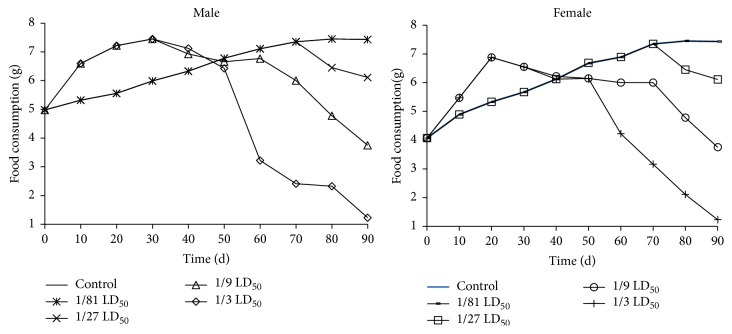
Food consumption changes for male and female mice for 90 days (*n* = 10). Data expressed as means ± standard deviation (SD).

No mortality was observed at any of the tested dose levels during the 90-day treatment period. At the beginning of the study, the mice in the 1/3 LD_50_ dose group showed increased food intake (Figure 2), whereas that of the other treatment groups was similar to the control group. After 60 days, a reduction in food intake and activities consisting of lethargy and asthenia were observed in the 1/3 LD_50_ dose group. After 70 days, the toxicity symptoms including cowering together, a loss of appetite, lethargy, slow reaction time, and reduced food intake were observed in the 1/9 LD_50_ dose group. After 80 days, a loss of appetite, lethargy, and a slow reaction time were observed in the 1/27 LD_50_ dose group.

**Figure 3:**
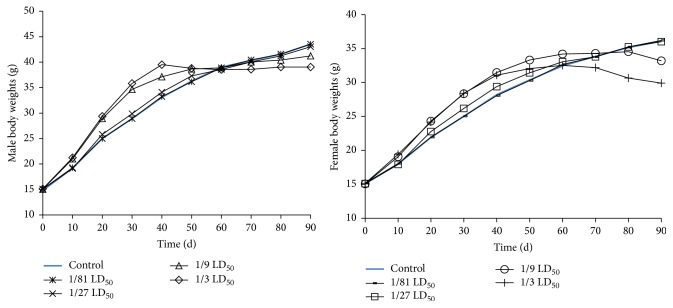
Body weights changes of male and female mice after 90-day treatment (*n* = 10). Data expressed as means ± standard deviation (SD).

Figure 3 shows that during the first 50 days, the weights of the 1/3 and 1/9 LD_50_ treatment groups increased rapidly. In addition, after day 50, the weights of the 1/3 LD_50_ group increased slowly and by day 80, the weights in the 1/9 LD_50_ treatment group increased slowly (*P* < 0.05). All the values were considered to be ethyl maltol complex-related due its toxicity.

### 3.3. Effect of Ethyl Maltol and Iron Complexes on Organ Index in Mice

The mean and relative organ weights for both male and female mice in this study were determined following the termination of the 90-day study and are presented in Table 2. Compared with the control group, the liver weights of the male and female mice in the 1/27 LD_50_ group had increased significantly by the end of the treatment period (*P* < 0.05). The kidney weights of the male mice in the 1/9 LD_50_ group increased significantly while those of females of the 1/27 LD_50_ group did not.

### 3.4. Effect of Ethyl Maltol and Iron Complexes on Haematological Parameters of Mice

Compared with the values of the control group mice, the Hb of the 1/27, 1/3, and 1/9 LD_50_ treatment group male mice differed significantly (*P* < 0.05) while that of the females in the 1/3 and 1/9 LD_50_ treatment groups did not. A significant increase in the RBC, WBC, N, and E counts was observed in the 1/9 LD_50_ treatment group male mice (*P* < 0.05). In addition, similar changes in these indices were observed in the 1/9 and 1/3 LD_50_ treatment group female mice.

The haematological parameters are shown in Tables 3 and 4. The Hb, RBC, and L of the mice of both sexes in the 1/27 LD_50_ treatment group were significantly decreased (*P* < 0.05), as were the WBC, N, and E of the 1/9 LD_50_ group (*P* < 0.05). All the significant differences observed were compared with those of the control group and are presented in Table 3.

### 3.5. Effect of Ethyl Maltol and Iron Complexes on Biochemical Parameters of Mice

Compared with the levels of the mice in the control group, the ALB, TBIL, ALT, ALP, AST, BUN, and creatinine levels of the 1/27 LD_50_ treatment group mice of both sexes were significantly different (*P* < 0.05). In particular, the TBIL, ALT, AST, BUN, and creatinine levels were increased in a dose-dependent manner relative to the control male and female mice, and the 1/9 LD_50_ and 1/3 LD_50_ treatment group values were statistically significant (Table 5). The LDH level of the male mice in the 1/27 LD_50_ treatment group was significantly increased (*P* < 0.05), while the level of the female 1/9 LD_50_ treatment group mice was significantly different (*P* < 0.05). All the changes reported as statistically significant at the study termination provide evidence of the effect of the ethyl maltol and iron complexes.

### 3.6. Effect of Ethyl Maltol and Iron Complexes on Histopathological Data in Mice

**Figure 4:**
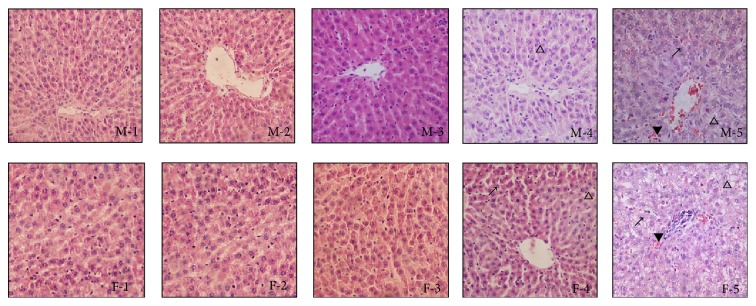
Histopathological examination of livers of male and female mice (haematoxylin and eosin, HE ×400) (*n* = 10). ↗ means granular denaturation, △ means vacuolar degeneration, and ▼ means haemorrhage. M-1, M-2, M-3, M-4, and M-5 were, respectively, from liver of male control, 1/81, 1/27, 1/9, and 1/3 LD_50_ treatment groups; F-1, F-2, F-3, F-4, and F-5 were, respectively, from liver of female control, 1/81, 1/27, 1/9, and 1/3 LD_50_ treatment groups.

**Figure 5:**
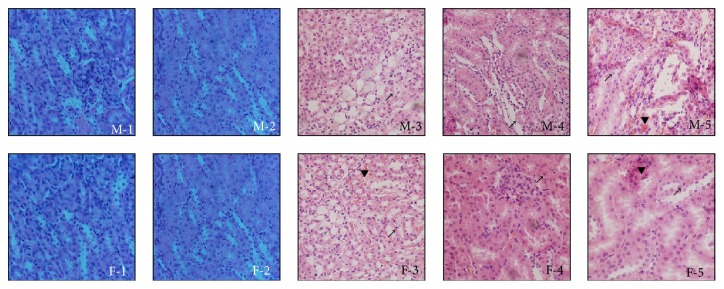
Histopathological examination of kidneys of male and female mice (haematoxylin and eosin, HE ×400) (*n* = 10). ↗ means granular denaturation; ▼ means haemorrhage; M-1, M-2, M-3, M-4, and M-5 were, respectively, from kidneys in male control, 1/81, 1/27, 1/9, and 1/3 LD_50_ treatment groups; F-1, F-2, F-3, F-4, and F-5 were, respectively, from kidneys in female control, 1/81, 1/27, 1/9, and 1/3 LD_50_ treatment groups.

Following treatment with the ethyl maltol and iron complexes, the kidney tissues of the male and female mice were histopathologically examined at the end of the 90-day administration period (Figure 4). Lesions were observed in the livers of the 1/9 and 1/3 LD_50_ treatment groups. The hepatocytes were swollen, and the cap between adjacent hepatocytes was not obvious. There were numerous tiny empty bubbles and protein grain showing a honeycomb or reticular pattern with nuclei suspended among them. The nuclei and the surrounding boundary were not clearly demarcated, and the presence of nuclear enrichment, fusion, membrane disappearance, and granular denaturation, as well as vacuolar degeneration, were also observed in the 1/3 LD_50_ treatment group (Figure 4(M-5)). The hepatocytes were swollen, and there was a disordered sinusoidal pattern with similar lesions in the 1/9 LD_50_ treatment group (Figure 4(M-4)), but the lesions and the colour of the hepatocytes were milder. Furthermore, lesions were also observed in the female mice in the treatment groups. However, compared to the male mice, the lobular architecture had disappeared, while numerous hepatocytes showed necrosis and a similar nuclear fusion was also observed in the female mice in the 1/3 LD_50_ treatment group (Figure 4(F-5)). Compared to the treated male mice, the lobular architecture disappeared, and the phenomena of granular denaturation and vacuolar degeneration were more serious in the female mice of the 1/9 LD_50_ treatment group (Figure 4(F-4)).

The kidneys showed evidence of swelling and the tissue sections were muddy. There were numerous RBC in the kidney tubular lumen while the renal epithelial cells were swollen, and granular denaturation, nucleus enrichment, and interstitial hemorrhage in renal tubule were observed with nuclear swelling in the 1/3 LD_50_ treatment group (Figure 5(M-5)). The renal epithelial cells were swollen and showed evidence of necrosis while granular denaturation was observed with lesions that narrowed the lumen, which showed the presence of a protein-like substance in the 1/9 LD_50_ group (Figure 5(M-4)). We observed similar lesions in the female mice in the treatment groups, but compared to the males the lesions in the 1/3 LD_50_ treatment group (Figure 5(F-5)) were more serious than those of the male mice. The tubular structure was disordered, the lumen had narrowed, the renal epithelial cells were swollen, and granular denaturation was also observed in the 1/9 LD_50_ group (Figure 5(F-4)). Finally, the renal epithelial cells were swollen and showed evidence of necrosis while granular denaturation, haemorrhage, and protein-like substance were evident in lumen of the 1/27 LD_50_ group (Figure 5(F-3)).

## 4. Discussion

It is well known that the resulting 1 : 3 (metal-ligand) complexes can partition readily across cell membranes and, thereby, may facilitate Fe transport across the intestinal wall [10]. Ethyl maltol also can hold Fe(III) in solution and subsequently donate it to an endogenous uptake system. In light of these points, we considered that the rapid growth of the 1/3 and 1/9 LD_50_ treatment groups was caused by the ethyl maltol and iron complexes while the decreased body weight was due to the accumulation of toxic ethyl maltol and iron complexes. Furthermore, the increase in the organ weights indicates the presence of organ hyperaemia, oedema, hyperplasia, and hypertrophy [11, 12]. In our study, the liver and kidneys weights consistently increased, which was in agreement with our theory that the ethyl maltol and iron complexes are poisonous. Furthermore, the changes in the body and organ weights were a dose-dependent increase.

Ethyl maltol combines with Fe^3+^ in different ratios, namely, 1 : 1, 1 : 2, and 1 : 3 [5], and it can combine with Fe^3+^ in the blood. Therefore, the lack of Fe^3+^ induced the dose-dependent decrease in the RBC count and Hb level while a dose-dependent increase in the WBC count was observed in the 1/3 LD and 1/9 LD_50_ treatment groups on D90. We considered that the haematological parameters change observed on D45 might be attributable to the unstable dynamics of the ethyl maltol and iron complexes. When the dose of ethyl maltol and iron complexes was 1/27 LD_50_, the complexes likely separated because of the unstable dynamic, and the haematological parameters increased as well. Collectively, these results provided strong evidence indicating the low risk of the ethyl maltol and iron complexes inducing toxicity following oral exposure.

ALT and AST, which exist mainly in the liver cells and rarely occur free in the blood, are important biological indicators of liver injury [13–15]. Ennulat et al. [16] and Adenuga et al. [17] also reported that ALT and TBIL were consistently identified as having the highest diagnostic accuracy for biliary injury with or without concurrent hepatocellular necrosis. However, ALT (Table 4) is considered a more specific and sensitive indicator of direct hepatocellular injury, and evidence shows that it is associated with negative diagnostic outcomes in humans when it is elevated in the serum concurrently with elevated serum levels of TBIL [17, 18]. In our study, the ALP and AST but not ALT and TBIL levels (Table 4) were also significantly elevated in the mice of both sexes in the 1/27 LD_50_ treatment group. Therefore, we excluded the negative outcomes and considered that the liver function was damaged by the ethyl maltol and iron complexes. creatinine (Table 4), which is an important biological indicator of the kidney function, is produced by muscle metabolism and is completely excreted in the urine [14, 19]. The increase in creatinine and BUN was caused by the degeneration of the tubular epithelial cells and lesions of the glomerulus while the kidney function was damaged [20]. We detected a dose-dependent increase in BUN and creatinine in both male and female mice and, therefore, concluded that the kidney function was disrupted by the ethyl maltol and iron complexes, which can, therefore, be considered poisonous. Furthermore, all the alterations in the biochemical parameters were caused by the toxicity of the ethyl maltol and iron complexes.

The kidney and liver lesions were clear [21–23]. In the 1/3 and 1/9 LD_50_ treatment groups, swollen hepatocytes, disordered sinusoidal pattern, granular denaturation, vacuolar degeneration, and nucleus fusion were observed. The 1/3, 1/9, and 1/27 LD_50_ treatment groups exhibited swollen renal epithelial cells, which were necrotic while granular denaturation was also observed. The lesions were dose- and sex-dependent, since those of the female mice were more serious than the males in the 1/3 and 1/9 LD_50_ groups. The lesions of the liver and kidneys were caused by the toxicity of the ethyl maltol and iron complexes, which are poisonous.

## 5. Conclusion

In summary, oral LD_50_ of the ethyl maltol and iron complexes was determined to be 743.88 mg kg BW^−1^ in mice. The ethyl maltol and iron complexes targeted the endocrine organs including the liver and kidneys following the 90 D oral exposure. Based on the haematological data, the lowest-observed-adverse-effect level (LOAEL) of the ethyl maltol and iron complexes was determined to be 1/81 LD_50_ (9.18 mg kg BW^−1^ day^−1^) in both male and female mice. Therefore, we suggest that alternative strategies for preparing the hot-pot, including the use of non-Fe-based cookware, need to be developed and encouraged to avoid the formation of the potentially toxic complexes.

## Figures and Tables

**Table 1:** Mortality rate in acute toxicity study in mice (*n* = 10).

Groups	Control group	1	2	3	4	5	6
Dose (mg kg^−1^)	0	425.98	532.48	665.6	832	1040	1300
Mortality rate	0	1	2	4	6	8	9
Mortality (%)	0	10	20	40	60	80	90
*P* ^2^	0	0.01	0.04	0.16	0.36	0.64	0.81

**Table 2:** Effect of ethyl maltol complexes on male and female mouse organ coefficients (*n* = 10).

Organs	Control group	Test groups			
		1/81 LD_50_	1/27 LD_50_	1/9 LD_50_	1/3 LD_50_
*Males*					
Heart	0.55 ± 0.01	0.55 ± 0.01	0.55 ± 0.01	0.55 ± 0.01	0.55 ± 0.01
Liver	5.33 ± 0.04	5.38 ± 0.03	5.48 ± 0.03^*∗*^	5.74 ± 0.04^*∗*^	5.73 ± 0.03^*∗*^
Spleen	0.46 ± 0.01	0.45 ± 0.01	0.46 ± 0.01	0.46 ± 0.01	0.46 ± 0.01
Lung	0.66 ± 0.01	0.67 ± 0.01	0.67 ± 0.01	0.67 ± 0.01	0.67 ± 0.01
Kidneys	1.46 ± 0.04	1.50 ± 0.02	1.55 ± 0.03	1.72 ± 0.04^*∗*^	1.74 ± 0.03^*∗*^
*Females*					
Heart	0.53 ± 0.01	0.53 ± 0.01	0.53 ± 0.01	0.52 ± 0.01	0.53 ± 0.01
Liver	5.26 ± 0.06	5.25 ± 0.06	5.61 ± 0.22^*∗*^	5.65 ± 0.06^*∗*^	5.75 ± 0.02^*∗*^
Spleen	0.44 ± 0.01	0.44 ± 0.01	0.44 ± 0.01	0.44 ± 0.01	0.44 ± 0.01
Lung	0.62 ± 0.01	0.62 ± 0.01	0.62 ± 0.01	0.62 ± 0.01	0.62 ± 0.01
Kidneys	1.20 ± 0.02	1.22 ± 0.02	1.30 ± 0.01^*∗*^	1.51 ± 0.02^*∗*^	1.50 ± 0.02^*∗*^

Results are expressed as means ± standard deviation (SD). ^*∗*^*P* < 0.05 versus the vehicle group.

**Table 3:** Haematological parameters of mice after 45-day treatment with ethyl maltol and iron complexes (*n* = 10).

Haematological indices	Control group	Treatment			
		1/81 LD_50_	1/27 LD_50_	1/9 LD_50_	1/3 LD_50_
*Males*					
Hb (g L^−1^)	146.09 ± 2.13	147.09 ± 1.99	152.25 ± 1.10^*∗*^	160.09 ± 0.76^*∗*^	156.97 ± 0.65^*∗*^
RBC (10^12^ L^−1^)	8.63 ± 0.22	8.53 ± 0.20	8.45 ± 0.22	9.22 ± 0.09^*∗*^	8.94 ± 0.09
WBC (10^9^ L^−1^)	7.58 ± 0.31	7.75 ± 0.34	7.49 ± 0.38	8.43 ± 0.17^*∗*^	7.77 ± 0.17
N	28.66 ± 0.41	28.88 ± 0.20	28.84 ± 0.10	29.45 ± 0.18^*∗*^	28.98 ± 0.13
E	2.91 ± 0.08	2.89 ± 0.08	2.95 ± 0.02	2.71 ± 0.07^*∗*^	2.80 ± 0.08
DC (%) B	0.14 ± 0.02	0.18 ± 0.01	0.17 ± 0.01	0.17 ± 0.01	0.17 ± 0.01
M	2.38 ± 0.11	2.39 ± 0.09	2.44 ± 0.08	2.41 ± 0.11	2.61 ± 0.04
L	65.90 ± 0.51	65.66 ± 0.23	65.60 ± 0.11	65.26 ± 0.18	65.43 ± 0.11
*Females*					
Hb (g/L)	131.53 ± 2.2	131.27 ± 2.12	133.92 ± 1.74	139.31 ± 1.30^*∗*^	137.27 ± 1.74^*∗*^
RBC (10^12^/L)	7.90 ± 0.08	7.85 ± 0.05	8.02 ± 0.05	8.58 ± 0.11^*∗*^	8.18 ± 0.08^*∗*^
WBC (10^9^/L)	5.88 ± 0.071	5.87 ± 0.09	6.00 ± 0.06	6.94 ± 0.06^*∗*^	6.22 ± 0.09^*∗*^
N	27.83 ± 0.52	27.98 ± 0.48	29.71 ± 0.29^*∗*^	33.16 ± 0.49^*∗*^	31.11 ± 0.08^*∗*^
E	3.13 ± 0.03	3.15 ± 0.03	2.99 ± 0.02^*∗*^	2.46 ± 0.07^*∗*^	2.76 ± 0.04^*∗*^
DC (%) B	0.91 ± 0.05	0.93 ± 0.03	0.90 ± 0.04	0.84 ± 0.06	0.91 ± 0.04
M	2.04 ± 0.03	2.06 ± 0.03	2.10 ± 0.03	2.36 ± 0.03	2.15 ± 0.03
L	66.09 ± 0.53	65.89 ± 0.50	64.30 ± 0.31	61.20 ± 0.55	63.06 ± 0.08

The results are expressed as means ± standard deviation (SD). Hb: haemoglobin; RBC: red blood cell; WBC: white blood cell; N: neutrophil; E: eosinophil; B: basophil; M: monocyte; L: lymphocytes. ^*∗*^*P* < 0.05, versus the vehicle group.

**Table 4:** Haematological parameters of mice after 90-day treatment with ethyl maltol and iron complexes (*n* = 10).

Haematological indices	Control group	Treatment			
		1/81 LD_50_	1/27 LD_50_	1/9 LD_50_	1/3 LD_50_
*Males*					
Hb (g L^−1^)	158.06 ± 1.03	158.16 ± 0.91	146.31 ± 0.74^*∗*^	124.60 ± 1.93^*∗*^	108.33 ± 3.13^*∗*^
RBC (10^12^ L^−1^)	10.33 ± 0.34	10.24 ± 0.25	8.37 ± 0.15^*∗*^	7.61 ± 0.15^*∗*^	6.35 ± 0.18^*∗*^
WBC (10^9^ L^−1^)	8.37 ± 0.23	8.12 ± 0.15	8.43 ± 0.16	10.18 ± 0.26^*∗*^	12.36 ± 0.25^*∗*^
N	28.68 ± 0.27	28.28 ± 0.23	29.20 ± 0.23	32.40 ± 0.37^*∗*^	34.56 ± 0.28^*∗*^
E	2.83 ± 0.05	2.86 ± 0.03	2.75 ± 0.03	2.52 ± 0.03^*∗*^	2.44 ± 0.04^*∗*^
DC (%) B	0.93 ± 0.03	0.93 ± 0.03	0.96 ± 0.03	0.91 ± 0.45	0.93 ± 0.02
M	2.19 ± 0.06	2.20 ± 0.04	2.18 ± 0.05	2.12 ± 0.03	2.15 ± 0.04
L	65.37 ± 0.23	65.73 ± 0.22	64.93 ± 0.23^*∗*^	62.05 ± 0.34^*∗*^	59.91 ± 0.26^*∗*^
*Females*					
Hb (g L^−1^)	140.87 ± 0.82	140.53 ± 0.78	134.98 ± 0.48^*∗*^	126.35 ± 1.08^*∗*^	115.56 ± 2.26^*∗*^
RBC (10^12^ L^−1^)	9.35 ± 0.17	9.71 ± 0.23	8.21 ± 0.18^*∗*^	7.08 ± 0.09^*∗*^	6.19 ± 0.11^*∗*^
WBC (10^9^ L^−1^)	7.93 ± 0.14	7.91 ± 0.11	8.28 ± 0.07	10.36 ± 0.28^*∗*^	12.34 ± 0.30^*∗*^
N	27.18 ± 0.38	27.50 ± 0.33	29.15 ± 0.32	32.33 ± 0.25^*∗*^	34.14 ± 0.17^*∗*^
E	2.88 ± 0.03	2.86 ± 0.03	2.75 ± 0.04	2.65 ± 0.04^*∗*^	2.42 ± 0.02^*∗*^
DC (%) B	0.97 ± 0.05	0.94 ± 0.02	0.95 ± 0.04	0.94 ± 0.03	0.95 ± 0.04
M	2.23 ± 0.05	2.18 ± 0.03	2.15 ± 0.05	2.11 ± 0.04	2.11 ± 0.03
L	66.73 ± 0.44	66.50 ± 0.31	65.19 ± 0.30^*∗*^	62.06 ± 0.23^*∗*^	60.55 ± 0.13^*∗*^

The results are expressed as means ± standard deviation (SD). ^*∗*^*P* < 0.05 versus the vehicle group.

**Table 5:** Biochemical parameters of mice of both sexes after 90-day treatment with ethyl maltol and iron complexes (*n* = 10).

Biochemical parameters	Control group	Treatment			
		1/81 LD_50_	1/27 LD_50_	1/9 LD_50_	1/3 LD_50_
*Males*					
TBIL (*µ*mol L^−1^)	1.93 ± 0.07	1.95 ± 0.06	2.17 ± 0.05^*∗*^	2.75 ± 0.06^*∗*^	3.04 ± 0.06^*∗*^
ALT (U L^−1^)	46.48 ± 2.40	46.16 ± 2.64	63.44 ± 2.45^*∗*^	86.19 ± 2.25^*∗*^	95.34 ± 2.82^*∗*^
ALP (U L^−1^)	111.54 ± 3.44	111.38 ± 2.93	96.12 ± 2.10^*∗*^	84.46 ± 2.0^*∗*^	74.42 ± 2.0^*∗*^
AST (U L^−1^)	145.23 ± 3.17	146.94 ± 2.85	159.78 ± 2.79^*∗*^	201.86 ± 3.44^*∗*^	236.58 ± 6.13^*∗*^
BUN (mmol L^−1^)	10.33 ± 0.31	10.49 ± 0.29	11.78 ± 0.34^*∗*^	14.05 ± 0.47^*∗*^	16.28 ± 0.41^*∗*^
creatinine (*μ*mol L^−1^)	13.60 ± 0.28	13.37 ± 0.45	14.88 ± 0.38^*∗*^	18.14 ± 0.38^*∗*^	19.45 ± 0.26^*∗*^
LDH (U L^−1^)	889.96 ± 4.48	888.80 ± 4.26	905.53 ± 3.35^*∗*^	970.73 ± 5.41^*∗*^	970.73 ± 5.41^*∗*^
*Females*					
TBIL (*µ*mol L^−1^)	1.70 ± 0.02	1.71 ± 0.01	1.80 ± 0.01^*∗*^	1.89 ± 0.02^*∗*^	2.15 ± 0.03^*∗*^
ALT (U L^−1^)	44.40 ± 4.21	46.09 ± 1.74	65.38 ± 3.18^*∗*^	71.62 ± 1.69^*∗*^	111.22 ± 4.44^*∗*^
ALP (U L)	143.09 ± 4.01	138.33 ± 2.20	112.14 ± 3.37^*∗*^	98.46 ± 3.62^*∗*^	78.51 ± 3.60^*∗*^
AST (U L^−1^)	142.71 ± 3.60	149.90 ± 1.09	178.61 ± 3.47^*∗*^	192.30 ± 1.60^*∗*^	215.21 ± 7.30^*∗*^
BUN (mmol L^−1^)	8.42 ± 0.31	8.63 ± 0.30	8.93 ± 0.19^*∗*^	10.64 ± 0.38^*∗*^	12.66 ± 0.25^*∗*^
creatinine (*µ*mol L^−1^)	11.16 ± 0.55	11.50 ± 0.49	12.99 ± 0.45^*∗*^	13.84 ± 0.28^*∗*^	14.71 ± 0.31^*∗*^
LDH (U L^−1^)	640.69 ± 6.49	637.72 ± 4.78	650.97 ± 4.87	670.83 ± 4.48^*∗*^	660.86 ± 5.15^*∗*^

Results are expressed as means ± standard deviation (SD). ^*∗*^*P* < 0.05 versus the vehicle group.
